# Chen, Zheng, *et al*. Anti-HIV Actions of 7- and 10-Substituted Camptothecins. *Molecules*, 2010, *15*, 138-148 

**DOI:** 10.3390/molecules15010149

**Published:** 2010-01-04

**Authors:** Yong-Tang Zheng

**Affiliations:** Key Laboratory of Animal Models and Human Disease Mechanisms of Chinese Academy of Sciences & Yunnan Province, Kunming Institute of Zoology, Chinese Academy of Sciences, Kunming, Yunnan 650223, China

The author list, affiliations and contact information of this paper [1] are revised to read as follows:

Yu-Ye Li ^1,2,3,†^, Ying-Qian Liu ^4,†^, Liu-Meng Yang ^1^, Rui-Rui Wang ^1^, Wei Pang ^1 ^, Shi-Wu Chen ^4 ^Xuan Tian ^4,^* and Yong-Tang Zheng ^1,^*

^1 ^Key Laboratory of Animal Models and Human Disease Mechanisms of Chinese Academy of Sciences & Yunnan Province, Kunming Institute of Zoology, Chinese Academy of Sciences, Kunming, Yunnan 650223, China

^2 ^Graduate School of the Chinese Academy of Sciences, Beijing 100039, China

^3 ^The First Affiliated Hospital of Kunming Medical College, Kunming, Yunnan 650032, China

^4 ^State Key Laboratory of Applied Organic Chemistry, Lanzhou University, Lanzhou 730000, China

^† ^ These authors contributed equally to this work.

* Authors to whom correspondence should be addressed; E-Mail: zhengyt@mail.kiz.ac.cn (Y-T.Z.); xuant@lzu.edu.cn (X.T.); Tel./Fax: +86 871 5195684 (Y.-T.Z).
